# Systems Analysis of Adaptive Responses to MAP Kinase Pathway Blockade in BRAF Mutant Melanoma

**DOI:** 10.1371/journal.pone.0138210

**Published:** 2015-09-25

**Authors:** Brian J. Capaldo, Devin Roller, Mark J. Axelrod, Alex F. Koeppel, Emanuel F. Petricoin, Craig L. Slingluff, Michael J. Weber, Aaron J. Mackey, Daniel Gioeli, Stefan Bekiranov

**Affiliations:** 1 Department of Biochemistry and Molecular Genetics, University of Virginia, Charlottesville, Virginia, United States of America; 2 Department of Microbiology, Immunology, and Cancer, University of Virginia, Charlottesville, Virginia, United States of America; 3 Bioinfomatics Core Facility, University of Virginia, Charlottesville, Virginia, United States of America; 4 Center for Applied Proteomics and Molecular Medicine, School of Systems Biology, College of Science, George Mason University, Manassas, Virginia, United States of America; 5 Department of Surgery, University of Virginia, Charlottesville, Virginia, United States of America; 6 Center for Public Health Genomics, University of Virginia, Charlottesville, Virginia, United States of America; The Moffitt Cancer Center & Research Institute, UNITED STATES

## Abstract

Fifty percent of cutaneous melanomas are driven by activated *BRAF* ^V600E^, but tumors treated with RAF inhibitors, even when they respond dramatically, rapidly adapt and develop resistance. Thus, there is a pressing need to identify the major mechanisms of intrinsic and adaptive resistance and develop drug combinations that target these resistance mechanisms. In a combinatorial drug screen on a panel of 12 treatment-naïve *BRAF* ^V600E^ mutant melanoma cell lines of varying levels of resistance to mitogen-activated protein kinase (MAPK) pathway inhibition, we identified the combination of PLX4720, a targeted inhibitor of mutated BRaf, and lapatinib, an inhibitor of the ErbB family of receptor tyrosine kinases, as synergistically cytotoxic in the subset of cell lines that displayed the most resistance to PLX4720. To identify potential mechanisms of resistance to PLX4720 treatment and synergy with lapatinib treatment, we performed a multi-platform functional genomics analysis to profile the genome as well as the transcriptional and proteomic responses of these cell lines to treatment with PLX4720. We found modest levels of resistance correlated with the zygosity of the *BRAF* V600E allele and receptor tyrosine kinase (RTK) mutational status. Layered over base-line resistance was substantial upregulation of many ErbB pathway genes in response to BRaf inhibition, thus generating the vulnerability to combination with lapatinib. The transcriptional responses of ErbB pathway genes are associated with a number of transcription factors, including *ETS2* and its associated cofactors that represent a convergent regulatory mechanism conferring synergistic drug susceptibility in the context of diverse mutational landscapes.

## Introduction

The BRaf kinase is activated by mutation from Valine to Glutamate at position 600 (V600E) in approximately 50% of cutaneous melanomas [1], resulting in uncontrolled activation of the MAPK pathway and expression of genes that are drivers of cell growth and migration [2,3]. The importance of *BRAF* ^V600E^ as an oncogenic driver is demonstrated by the rapid response of most *BRAF* ^V600E^ mutant melanomas to the BRaf inhibitors vemurafenib or dabrafenib [4,5]: Over 50% of *BRAF* ^V600E^ mutant melanomas showed objective response after treatment [4,5].

Unfortunately, response to vemurafenib is not durable, with most patients relapsing within 2 to 18 months of initial treatment [6–8]. Several mechanisms of acquired resistance have been identified in melanoma that has relapsed after treatment. For example, exogenous *MITF* overexpression has been shown to repress the ability of BRaf inhibition to kill melanoma [9,10] and in cells that display high *MITF* activity, class III and class IV melanosomes can sequester drugs [11]. In more recent studies, lack of *MITF* activity has been implicated as an indication of resistance to BRaf inhibition [12,13]. Finally, a host of genomic alterations have been identified that circumvent the targeted inhibition of BRaf, in most cases reactivating the MAPK pathway: *BRAF* splice variants facilitate dimerization with *RAF1* and result in *MEK* activation [14]; *MEK* can be activated by mutation or by activation of *COT* [15]; the cytotoxic effects of MAPK pathway inhibition can be blunted by compensatory pathway activation, such as *PI3K* activation [16]; and the zygosity of the V600E mutation is associated with modulating response to treatment with vemurafenib [17–19]. Less is known about mechanisms of intrinsic or adaptive resistance that can be manifested within a few hours or days of treatment, and is the focus of the current investigation.

Combination therapies are predicted to overcome intrinsic, adaptive and acquired resistance [16]. For resistance acquired following relapse, DNA sequencing has uncovered mutational changes underlying resistance, and created the possibility for targeted combination therapies. However, there has been no systematic methodology established to predict effective combinations for newly diagnosed disease because of the complexity of the genetic changes in melanoma [16,20] and the consequent diversity of compensatory survival adaptations. Therefore, we and others [21] have taken an empirical approach, performing high-throughput combinatorial screens of drugs and tool compounds to identify the most effective combinations of drugs or pathways for more durable melanoma treatment. We screened a panel of 12 *BRAF* ^V600E^ mutant melanoma cell lines, with a wide range of intrinsic sensitivity to the BRaf inhibitor PLX4720, in combination with a library of 60 secondary drugs and tool compounds. Notably, Held et al. [21] found PLX4720 combined with lapatinib, an inhibitor of ErbB family receptor tyrosine kinases, was highly synergistic in a number of *BRAF* ^*V600E*^ melanoma cell lines. We also found that the 6 cell lines that were most resistant to PLX4720 displayed synergistic cytotoxicity with lapatinib. In order to determine mechanisms of resistance to PLX4720 and synergy to lapatinib as well as help develop systematic approaches to better predict which combinations might be effective/synergize, we performed a functional genomics and genetics profiling of the12 melanoma cell lines. Novel results from our study include coupling the roles of mutant *BRAF* zygosity and mutations in RTKs in determining basal drug resistance to broad up-regulation of ErbB pathway genes including ErbB family RTKs in response to PLX4720 treatment. Further analysis revealed enrichment of transcription factors including ETS family members and their associated co-factors as likely regulatory drivers of adaptive PLX4720 resistance, providing a potential convergence point of adaptive resistance within the diversity of response mechanisms.

## Results

### Analysis overview

**Fig 1:**
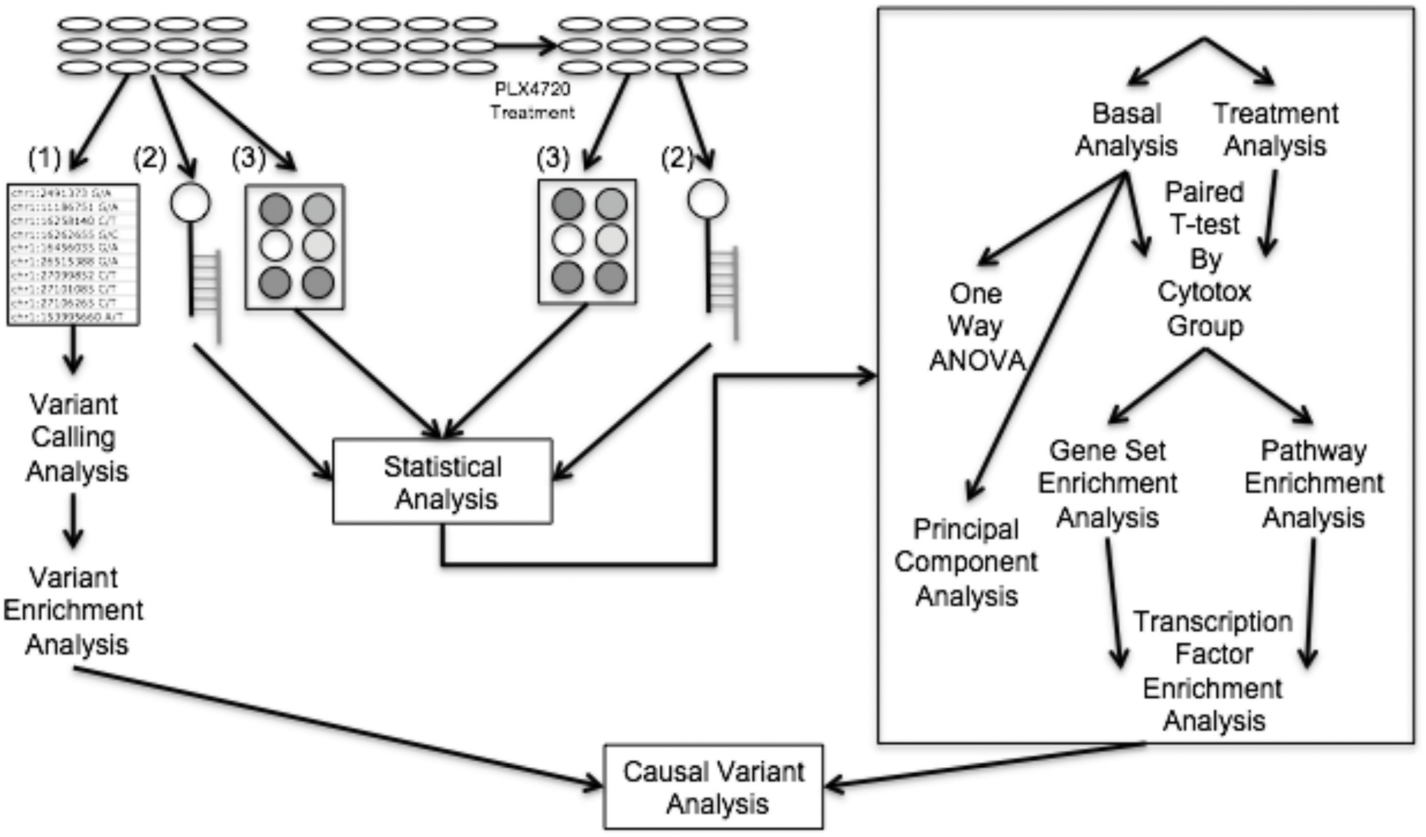
Functional genomic data generated and analysis workflow. Flow-chart including transcriptome, proteome and exome data generated and integrative analysis of these data sets. 12 cell lines (not drug-treated) were characterized using exome sequencing (1), gene expression arrays (2), and reverse phase protein arrays (3). Variant calling and enriched gene variants were identified by cross referencing our results with mutated genes associated with cancer identified by Lawrence et al [20]. Cell lines were exposed to PLX4720 and their responses were assayed with gene expression arrays and reverse phase protein arrays. Protein response to treatment was correlated with cytotoxic effects of PLX4720 treatment to identify proteins that might be mitigating the cytotoxic response. Cytotoxicity (Cytotox) groups were identified by clustering the cytotoxicity data. Differential gene expression responses to treatment within each cytotoxicity group were identified and underwent both gene set and pathway enrichment analysis via MSigDB and Pathway Express respectively. Pathway enrichment analysis revealed ErbB signaling as a key response to treatment and gene set enrichment analysis revealed a number of transcription factors that are enriched which putatively regulate ErbB signaling pathway genes.

In order to gain insights into the mechanisms of synergy and sensitivity, and potentially to identify clinically relevant biomarkers, we broadly profiled our panel of lines with multiple functional genomic and genetic assays (Fig 1). Analysis of the basal (i.e., untreated cellular state) transcriptome revealed differences in *MITF* expression level that correlated weakly with drug sensitivity. Dividing the cell lines into groups based on unsupervised clustering of all the single drug and combination cytotoxic responses across a three by three dose response matrix yielded five phenotype groups. Strikingly, these cytotoxicity groupings closely mimicked the groupings observed in the basal transcriptome based on a principal component analysis (PCA). The transcriptional and proteomic responses to PLX4720 treatment were then analyzed to identify molecular responses that were common between the cell lines in each group. The lists of differentially expressed genes and phosphoproteins were subjected to the Mutational Signatures Database (MSigDB) [22] enrichment analysis and Kyoto Encyclopedia of Genes and Genomes (KEGG) pathway enrichment analysis using Pathway Express [23] to identify transcription factors that putatively regulate the genes in the pathways associated with response to PLX4720 and synergy to PLX4720 and lapatinib (S1 Fig).

### Analysis of the basal transcriptome yields groupings based on *MITF* and drug synergy

**Fig 2:**
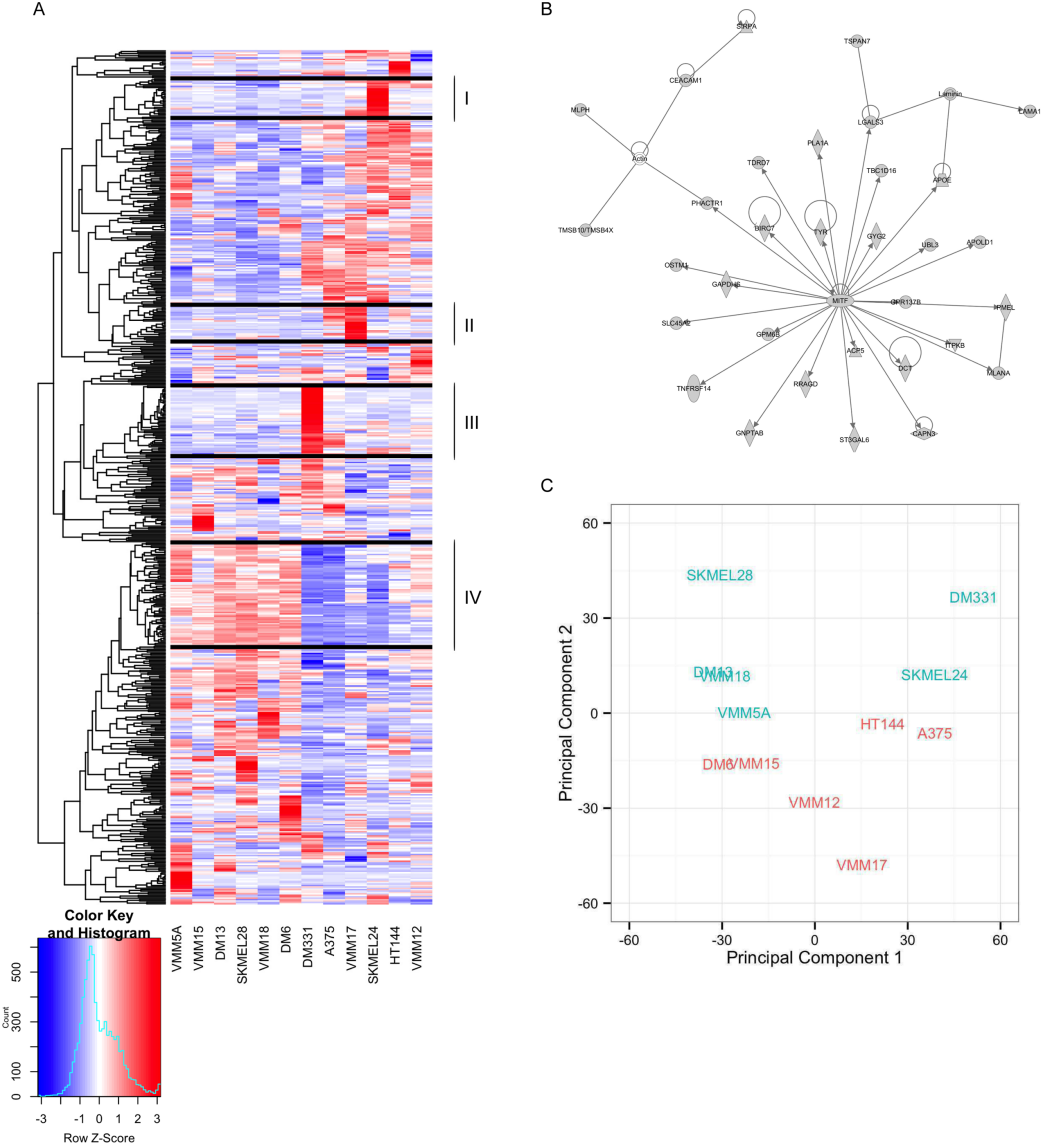
Clustering and PCA analysis of basal gene expression reveals *MITF* expression and gene regulation separates melanoma cell lines. We performed a one-way analysis of variance (ANOVA) and applied a 0.1% false discovery rate (FDR) cutoff to identify genes with significantly varying expression levels, and the genes (y-axis) and cell lines (x-axis) were organized by unsupervised hierarchical clustering. Clustering the genes using the correlation distance and average linkage yielded 91 gene clusters with the number of genes in each cluster ranging from 1 to 73 when applying a cluster height cutoff of 0.41. Hierarchical clustering of samples (x-axis) by genes (y-axis) of gene expression for significantly varying genes across twelve melanoma cell lines (A). The largest cluster (cluster IV), which contains *MITF* along with three others that contain cell line specific signatures (clusters I, II and III) are highlighted as they were the only clusters with greater than 20 genes. Cluster IV genes were analyzed using Ingenuity Pathway Analysis revealing the cluster contains *MITF* as a regulatory hub and its target genes (B). Principal component plot of basal expression across the twelve cell lines (C) separates cells according to *MITF* expression along the first principal component (i.e., decreasing *MITF* expression going from left to right) and Bliss score across the second principal component.

To determine whether the transcriptional profile of treatment-naïve cells could predict sensitivity to the drugs, singly or in combination, we classified the 12 cell lines based on unsupervised clustering of the basal (not drug-treated) transcriptome (Fig 2A). Clusters I and II contained genes that were relatively high-expressed only in SKMEL24 and VMM17, respectively, and, consequently, were not driving the ordering of the cell lines.

Cluster III (49 genes) contained relatively highly expressed genes in DM331, which is of interest because it is the most resistant line to PLX4720 treatment. A subset of the genes in this cluster was also relatively high-expressed in A375, our second most sensitive line to PLX4720 treatment. To identify the potential functional significance of the genes within cluster III, we performed a MSigDB enrichment analysis using the transcription factor gene set (S1 Table). The top result revealed a set of 9 genes (*TBL1X*, *IL6*, *CST7*, *NRG1*, *CPA4*, *SERPINB7*, *STC2*, *CALB2*, *RAB27B*) that all contain the *JUN* motif in their promoter. Among these genes, *TBL1X* and *CALB2* were the only two that were expressed at a relatively high level in both A375 and DM331 while the rest including *NRG1* were highly expressed in DM331 only. We note that exposure to *NRG1* has been shown to induce resistance to PLX4720 treatment in A375 cells [24]. Despite the fact that DM331 and A375 are at opposite ends of the range of sensitivity to PLX4720, their transcriptional profiles are strikingly similar—placing them next to each other in the clustering of cell lines (Fig 2A). Taken together, these results suggest that high expression of *NRG1* in DM331 contributes substantially to DM331’s intrinsic resistance to PLX4720 treatment.

Clustering the expression profile of the 73 genes in Cluster IV separates our cell lines into two distinct groups. Ingenuity Pathway analysis of this list of 73 genes revealed that cluster IV contained *MITF* as a hub with 32 *MITF* target genes (i.e., 45% *MITF*-associated genes in cluster IV) of which 32 are shown in Fig 2B. This suggests that the cell lines could be in different cell lineage states with the *MITF* transcriptional program driving this separation. To confirm whether *MITF* is a regulatory hub of cluster IV genes, we compared the genes in cluster IV with *MITF* target genes identified by Strub et. al. via a ChIP-Seq experiment [25] and observed an overlap of 33 of 73 (45%; p-value = 3.42 e-11; S2 Fig). These results implicate *MITF* and the expression of its associated targets as potentially driving lineage separation in our cell lines, but not contributing consistently to drug sensitivity.

To assess the extent to which the *MITF* expression is associated with response to PLX4720 treatment in our panel, we correlated *MITF* expression in each of our lines with their respective IC50 values. We observed a modest anticorrelation of -0.40 between the *MITF* expression level of our lines, and their respective IC50 doses, consistent with Konieczkowski et al. [12]. In our case, this result is largely driven by DM331, our most resistant line. When this line is excluded, we observe essentially no correlation, 0.085, between *MITF* gene expression and IC50 across the remaining eleven cell lines. Because our synergy studies were performed over a dose range that included concentrations below the IC50 (centered around IC20), we also examined the correlation of *MITF* expression and cytotoxicity at the low, medium and high doses. We found a relatively strong anticorrelation between *MITF* gene expression level and PLX4720 cytotoxicity (excluding DM331): -0.79, -0.68 and -0.67 for our low, medium and high doses, respectively. Notably, our PLX4720 doses, which were intended to assess drug synergy, are much lower, 125 nM, in our studies compared to other studies which tend to use ~1μM PLX4720 [11–13,19]. Thus, at low doses of PLX4720, we find lower levels of *MITF* across our cell lines are associated with increased sensitivity to PLX4720 treatment while at IC50 doses, we find essentially no correlation.

To analyze mechanisms of synergy between PLX4720 and lapatinib, we performed a principal components analysis (PCA) which reveals dominant sources of variability in the first principal component as well as sub-dominant sources of variability in the second and higher order principal components. As shown in the PCA plot (Fig 2C), the separation along the first principal component (PC1) was not associated with synergy, but appeared to be strongly correlated with *MITF* expression level in our lines (correlation coefficient = -0.9; S2 Table). We observed an inverse relationship between *MITF* expression level and *AXL* kinase expression level (correlation coefficient = -0.84 S3B Fig), a trend which has been observed previously [10,12]. The separation that we observed along the second principal component (PC2) segregated synergy and non-synergy cell lines, and was highly correlated with the synergy score of the lines (correlation coefficient = 0.791).

The strong correlation of the second principal component with the Bliss synergy score of the cell lines raised the possibility that the basal transcriptional profile could yield insights into mechanisms of synergy and resistance. To explore this possibility, we correlated each gene expression profile across cell lines with PC2 (S3 Table) and identified pathways enriched for these genes using MSigDB enrichment analyses [22]. We input genes whose correlation with PC2 was greater than 0.8 or less than -0.8. Pathways that were enriched with genes that were highly correlated with PC2 (S4 Table) were cell cycle and G-protein coupled receptor (GPCR) signaling associated pathways. Pathways that were enriched with genes that were highly anticorrelated with PC2 also included GPCR signaling, neuroligand GPCRs and cytokine-cytokine receptor interaction.

### Grouping cell lines according to their cytotoxicity profiles is consistent with their transcriptome-based grouping

**Fig 3:**
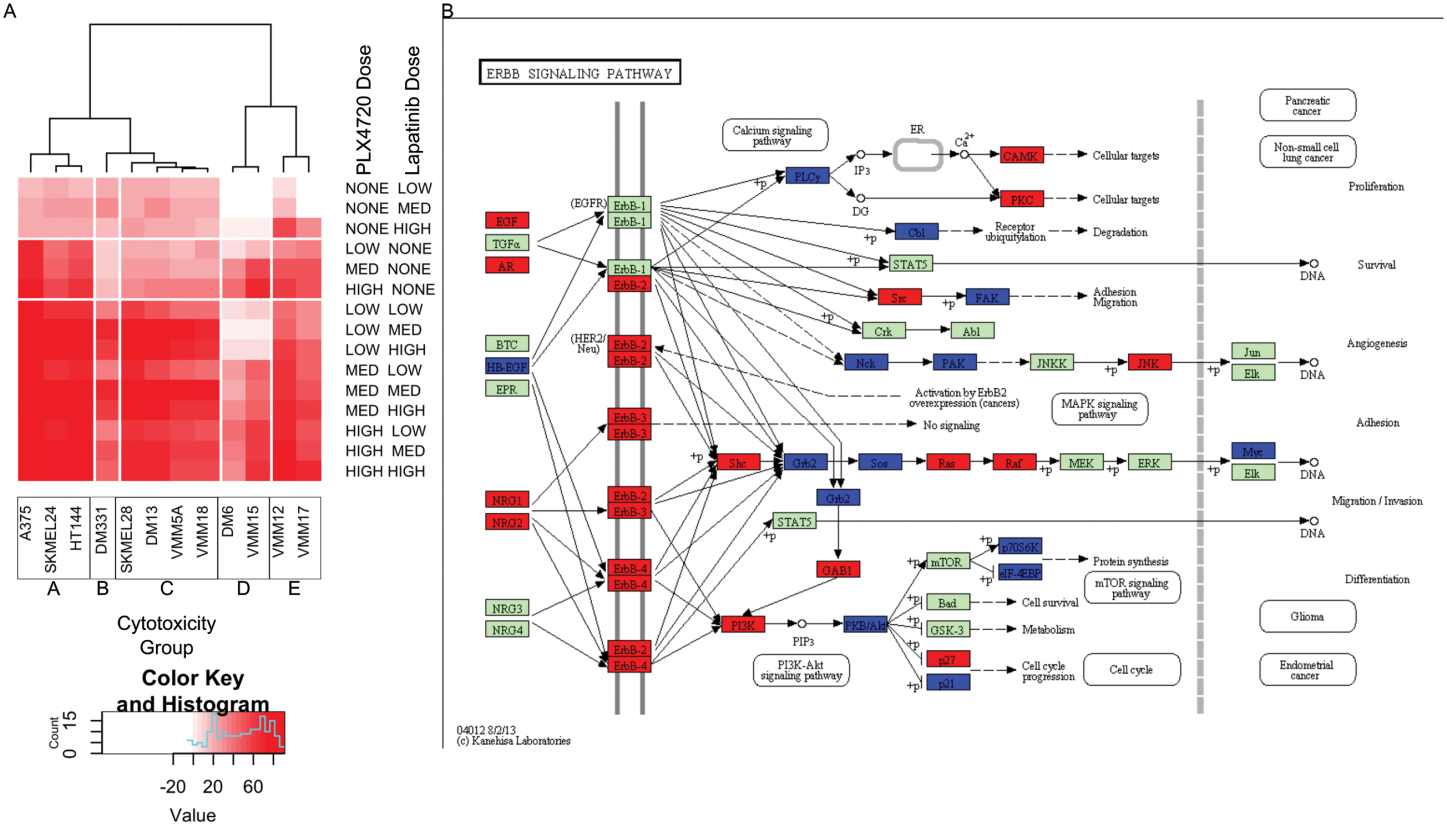
Clustering by cytotoxicity reveals differential responses to PLX4720 treatment. Clustering of cell lines (x-axis) according to cytotoxic responses to PLX4720, lapatinib, and combination treatment at three doses for each individual drug and a three by three dose response for the combination (y-axis) (A). Five groups were identified: CGA (most left, 3 lines), CGB (near left, 1 line), CGC (center, 4 lines), CGD (near right, 2 lines), and CGE (far right, 2 lines). Pathway enrichment analysis of differential gene expression to PLX4720 treatment identified *ERBB* signaling as being broadly activated in CGC (B). Refer to S6 Table for fold change values used to generate pathway diagram.

Given the modest association between basal gene expression and synergy, we wanted to determine if there was a phenotypic classification that better correlated with the basal gene expression analyses. We re-examined the grouping of the cell lines according to cytotoxic drug response over the low dose ranges used to identify synergy. These analyses of cytotoxic responses to PLX4720, lapatinib and the combination of the two drugs revealed that the lines that did not demonstrate synergy had low synergy scores for fundamentally different reasons (detailed below). To organize the cell lines according to drug cytotoxicity profiles, we clustered the cell lines using their cytotoxicity values across drug treatments (Fig 3A), again applying the Pearson correlation distance and average linkage. This cytotoxicity based clustering yielded five distinct phenotypic groups. The clustering of cell lines’ cytotoxic responses revealed a strong separation between the synergistic lines and the non synergistic lines with the exception of SKMEL24, a synergistic line which grouped with the non synergistic lines. To verify the robustness of the clustering in which averages of cytotoxicity values across replicates were used, we generated box plots of all the cytotoxicity values (S4 Fig). Both the trends with drug dose and actual cytotoxicity values were robustly similar across cell lines within a cytotoxic group and noticeably distinct between groups. Strikingly, cell lines within cytotoxic groups clustered according to their basal transcriptome-based grouping in the principal component plot (S3 Fig). The five groups identified were: (1) Cytotoxicity group A (CGA), whose cells show strong dose dependence on and relatively high cytotoxicity in response to PLX4720 treatment while lapatinib treatment is static across all doses, and its cytotoxicity is moderate; (2) Cytotoxicity group B (CGB), which only contains DM331. This cell line is almost completely resistant to PLX4720 in cell culture, and has relatively high expression of *NRG1*, a known mechanism of PLX4720 resistance [24]. For this reason, we exclude it from further transcriptional group analyses; (3) Cytotoxicity group C (CGC), in which cells respond to the drug combination but are resistant to either drug alone; (4) Cytotoxicity group D (CGD), in which cells grow in the presence of low dose lapatinib and have reduced response to combination at low doses compared to other groups; and (5) Cytotoxicity group E (CGE), whose cells grow in the presence of low dose lapatinib.

### Exome sequencing reveals mutations that modulate lineage-based cytotoxicity profiles

**Fig 4:**
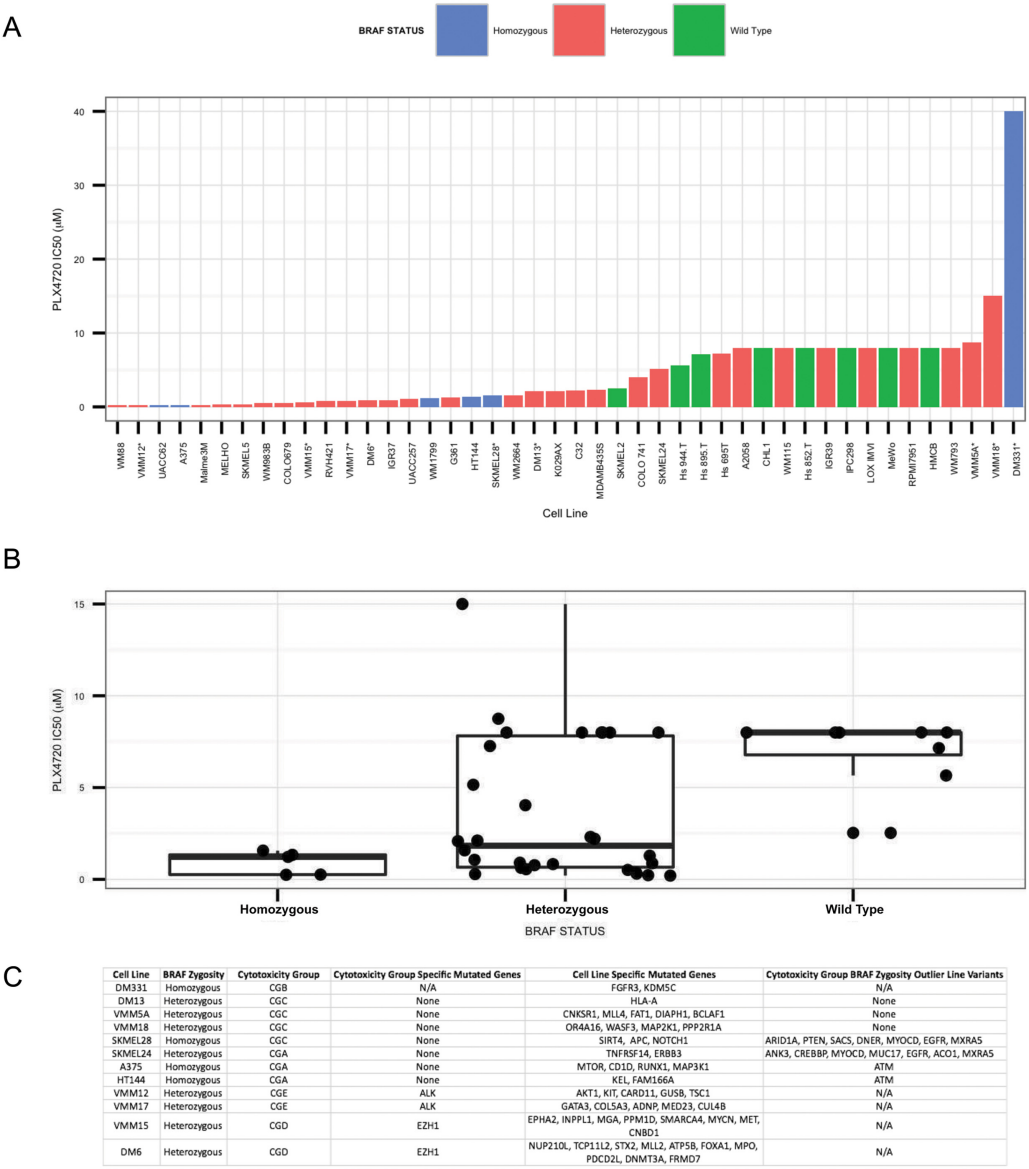
Exome analysis reveals trend between zygosity of V600E allele in the *BRAF* locus and sensitivity to PLX4720. Ranked IC50 values for *BRAF* ^*V600E*^ melanomas from our panel and the CCLE. For lines in our panel that were also in the Cancer Cell Line Encyclopedia [26] (CCLE), we used the IC50 values from the CCLE. Lines from our panel are denoted with “*”. Zygosity at the *BRAF* locus was assayed using IGV visualization of BAM files. Lines found to be homozygous for the V600E allele were colored blue, lines found to be heterozygous for the V600E allele were colored red, and lines with only wild type *BRAF* were colored green (A). Box plots of IC50 values for *BRAF* ^*V600E/V600E*^ (Homozygous), *BRAF* ^*WT/V600E*^ (Heterzygous), and *BRAF* ^*WT/WT*^ (Wild Type), melanoma cell lines (B). Exome sequencing identifies potential causal variants. Variants present in our lines were compiled using exome sequencing and cross referenced with genes identified as being mutated across multiple cancer types [20]. *BRAF* zygosity, genes uniquely mutated in each drug cytotoxicity group, and the genes uniquely mutated in each line are listed (C).

To determine whether the mutational landscape of the cell lines could explain their grouping according to cytotoxicity profiles, we performed whole exome sequencing on all twelve cell lines. We found a trend between the zygosity status of the *BRAF* locus and response to treatment, with homozygous *BRAF* mutant lines being more sensitive to BRaf inhibition, as has previously been reported in a panel of cell lines by Sondergaard et al [19]. The only overlapping line between their study and ours was SKMEL28, and we confirm the mutations that they observed. To expand this analysis, we obtained IC50 values to PLX4720 for 35 melanoma cell lines in the Cancer Cell Line Encyclopedia [26] and plotted the IC50 for each line in order, as well as lines in our study, and colored the bars based on their zygosity status at the *BRAF* locus (Fig 4A). We observed a trend between the zygosity and IC50, with five out of six homozygous lines located in the left (more sensitive) half of the chart. The only outlier was DM331, which we again excluded from our analysis. We obtained a p-value = 0.049 using a permutation-based t-test comparing IC50 values of the *BRAF* ^*V600E*^ homozygous lines versus the *BRAF* ^*V600E*^ heterozygous lines. We then generated box plots for the IC50 values for *BRAF* ^*V600E/V600E*^ melanomas, *BRAF* ^*WT/V600E*^ melanomas and *BRAF* ^*WT/WT*^ melanomas. We observed a clear trend between the zygosity status and the sensitivity to PLX4720 treatment (Fig 4B), similar to the findings of Halaban et al. [27]. Nevertheless, the most sensitive cell lines were *BRAF* ^*WT/V600E*^ melanomas. We hypothesized that these might contain one or more mutations that shunt the adaptive response to PLX4720 treatment. Consistent with this, the most PLX4720 sensitive line, WM88, contained a splice variant in ERBB4. We predict that there are likely similar mutations which could shunt the adaptive response to PLX4720 treatment in the other twelve relatively PLX4720 sensitive heterozygous cell lines. Lastly, we note that our study has a much larger percentage of *BRAF* ^*V600E/V600E*^ melanomas compared to other studies, which could explain why *BRAF* zygosity had a stronger association with sensitivity to PLX4720 treatment in our study compared to others.

We then searched for genes that contained variants that were unique to individual cytotoxicity groups and could be dominant drivers of drug sensitivity within each group. We found that lines in both the CGE and CGD groups contained unique genetic variants. Variants previously identified by dbSNP were removed, and we further filtered the list by identifying novel nonsynonymous variants; nonsense and missense variants only. The list of 10,000 variants was narrowed to 143 variants across 83 genes that were identified as being known drivers of cancer [20]. We imported the list of variants into polyphen2 [28] (S5 Table), which identifies variants that may alter function. We identified cytotoxicity group specific mutations in *ALK* and *EZH1* that were predicted to be damaging by polyphen2 in CGD and CGE cell lines, respectively. *ALK* is a receptor tyrosine kinase that has previously been associated with multiple cancer types [8,20,29] and is an activating mutation in many types of lung cancer [8]. *EZH1* is a histone methyltransferase that deposits methyl groups on histone H3 lysine 27 (H3K27me3), which tends to yield repressed chromatin [30]. *EZH1* has been shown to maintain stem cell identity and pluripotency [30]. Importantly, *EZH1* and *EZH2* have been identified as candidate oncogenes in diffuse large B cell lymphoma [31].

While we found no cytotoxicity group specific variants in CGA and CGC cell lines, *BRAF* zygosity potentially explains the response of three of the four CGC and two of the three CGA cell lines to PLX4720 treatment respectively. Consequently, we searched for genes that contained variants specifically in the cell lines that were cytotoxicity group exceptions with regard to BRAF zygosity (SKMEL28 and SKMEL24) that were not shared by other group members. We also identified variants that were specific to SKMEL28 and SKMEL24, which could explain their being placed in CGC and CGA groups, respectively.

In SKMEL28, which was the second most PLX4720 resistant homozygous cell line among the six shown in Fig 4B, we found several genes that could explain its increased resistance to PLX4720 treatment compared to other homozygous *BRAF* lines. SKMEL28, uniquely in our panel, contains a mutation in *NOTCH1*, a transmembrane protein that has been shown to interact with membrane bound ligands [32] and it can also activate MAPK signaling and PI3K signaling in melanoma [33]. SKMEL28 also contains notable variants in three tumor suppressors. *ARID1A* and *PTEN* are mutated in SKMEL28 but not in its cytotoxicity group members. However, *ARID1A* is also mutated in VMM15, and *PTEN* is not mutated in any other line. *ARID1A* is a *SWI/SNF* family member and potential tumor suppressor in melanoma [34]. Lastly, SKMEL28 is the only line in our panel with a mutation in *APC*, a tumor suppressor that antagonizes *WNT* signaling [20] and has been shown to be nonfunctional in patients with melanoma [35]. We hypothesize that this set of mutations in growth pathways and tumor suppressors potentially explains SKMEL28’s resistance to PLX4720 treatment, despite its homozygous *BRAF* status.

In SKMEL24 we found a number of cell surface proteins that contain mutations including *TNFRSF14*, *DNER*, *ERBB3* and *EGFR* (Fig 4C). A loss of function mutation in any of these proteins could limit SKMEL24 in its ability to activate alternative growth pathways in the presence of BRaf inhibition, thereby, potentially explaining its sensitivity to PLX4720 treatment despite its heterozygous mutant *BRAF* status. When considering its set of cell line-specific variants, the *ERBB3* mutation is notable given that ErbB family members are lapatinib targets. However, polyphen2 predicted this variant to be benign, with the 1077^th^ residue substituting an arginine for a tryptophan, so more analyses are required to determine the significance of this variant.

Finally, we applied our search for genes that contained variants in only one cell line in order to identify variants that could be driving cell line specific cytotoxicity responses. DM331 is our most aberrant line in that it contains homozygous *BRAF* ^*V600E*^, yet it is by far the most PLX4720 resistant cell line. We found that *FGFR3*, which is known to activate RAS family members [36],had a potential function altering-mutation in the kinase domain as identified by polyphen2 (i.e., substituting an arginine (R) for a lysine (K) at residue 618; S5 Table). We also previously observed its high expression of *NRG1*, a known contributor to PLX4720 resistance [24] and find that its basal expression of *EGFR* is far higher than is seen in the other lines.

Taken together, we find evidence for the hypothesis that the sensitivity of the lines to PLX4720 treatment can be explained in part by a combination of zygosity at the *BRAF* locus and the mutational status of various cell surface receptors.

### Cytotoxic group-wise analysis after PLX4720 treatment reveals broad ErbB pathway gene up-regulation in combination sensitive cell lines only

Because basal gene expression analysis revealed modest associations with drug sensitivity, we determined whether PLX4720 sensitivity and synergy with lapatinib could be due to changes in gene expression induced by PLX4720 treatment. To test this hypothesis, we generated and analyzed transcriptional and proteomic profiles for each of the cell lines 8 hours after PLX4720 treatment. Given the robust grouping of the cell lines according to cytotoxic drug response, we performed a group-wise analysis in order to gain power to detect subtle but consistent changes in gene expression in response to PLX4720. Specifically, we used a paired t-test within cytotoxicity groups on the transcriptional response to PLX4720 treatment matching each treatment sample with a control (i.e., no drug treatment) and applying a stringent 1% FDR cutoff (S6 Table). The CGA, CGC, CGD and CGE groups (Fig 3A) showed 2614, 7310, 2383, and 2993 probes detecting differential gene expression at a 1% FDR, respectively. When each of these gene lists was put through a Pathway Express [37,38] enrichment analysis, the top two pathways in all four groups were always leukocyte transendothelial migration and cell adhesion molecules, and the third ranked pathway in every group except CGC was adherens junctions (S7 Table). Only CGC—the group requiring both lapatinib and PLX4720 for maximal cytotoxicity—had upregulation of the noncanonical WNT and MAPK signaling pathways, which was not observed in the other groups based on a Bonferroni corrected gamma p-value of 0.01. Notably,CGC and CGA have significant Bonferroni corrected p-values for the ErbB signaling pathway (5.60e-05 and 4.86e-04 respectively). Visualization of differentially expressed genes in the ErbB signaling pathway across cytotoxicity groups gave the most insight into a potential mechanism for synergy and resistance (Fig 3B and S5 Fig). In CGC, there is broad upregulation (red) of the ErbB signaling pathway. Only in the CGC group do we observe the upregulation of multiple ErbB family members. In CGA and CGD, only *ERBB2* is upregulated, and in CGE the ErbB family is not significantly affected by treatment at all. Consistent with this result, Abel et al. have previously observed *ERBB3* activation in response to vemurafenib treatment [18]. When we looked at the pathways in cancer diagrams (S6 Fig), we observed that in addition to the differences in ErbB signaling pathway, WNT signaling (S7 Fig), and MAPK signaling (S8 Fig) were broadly affected in CGC only. In addition to the enrichment in ErbB signaling in CGA, we also observe significant enrichments in apoptosis, a number of cancer specific pathways, and p53 signaling pathways.

Given the striking, simple result that the ErbB pathway is broadly upregulated in response to PLX4720 treatment only in the group of cell lines that require treatment with the PLX4720 and lapatinib combination in order to achieve an optimally cytotoxic response, we asked which transcription factors were likely activated by mutant BRaf inhibition and targeting ErbB pathway genes. Using MSigDB [22], we identified transcription factors whose motif occurrence is significantly enriched at the promoters of genes that were differentially expressed in each cytotoxicity group (S8 Table). We found 45 unique transcription factor binding motifs that are only present in CGC, among which were an ETS family member and its associated cofactors, including *ETS2*, *GATA1*, and *POU2F1*. Additionally, we saw a number of well-established oncogenic transcription factor binding motifs, including *JUN* and *MYB*. Lastly, based on ChIP-seq data [25], we found that *MITF* targets a number of ErbB pathway genes [39].

**Fig 5:**
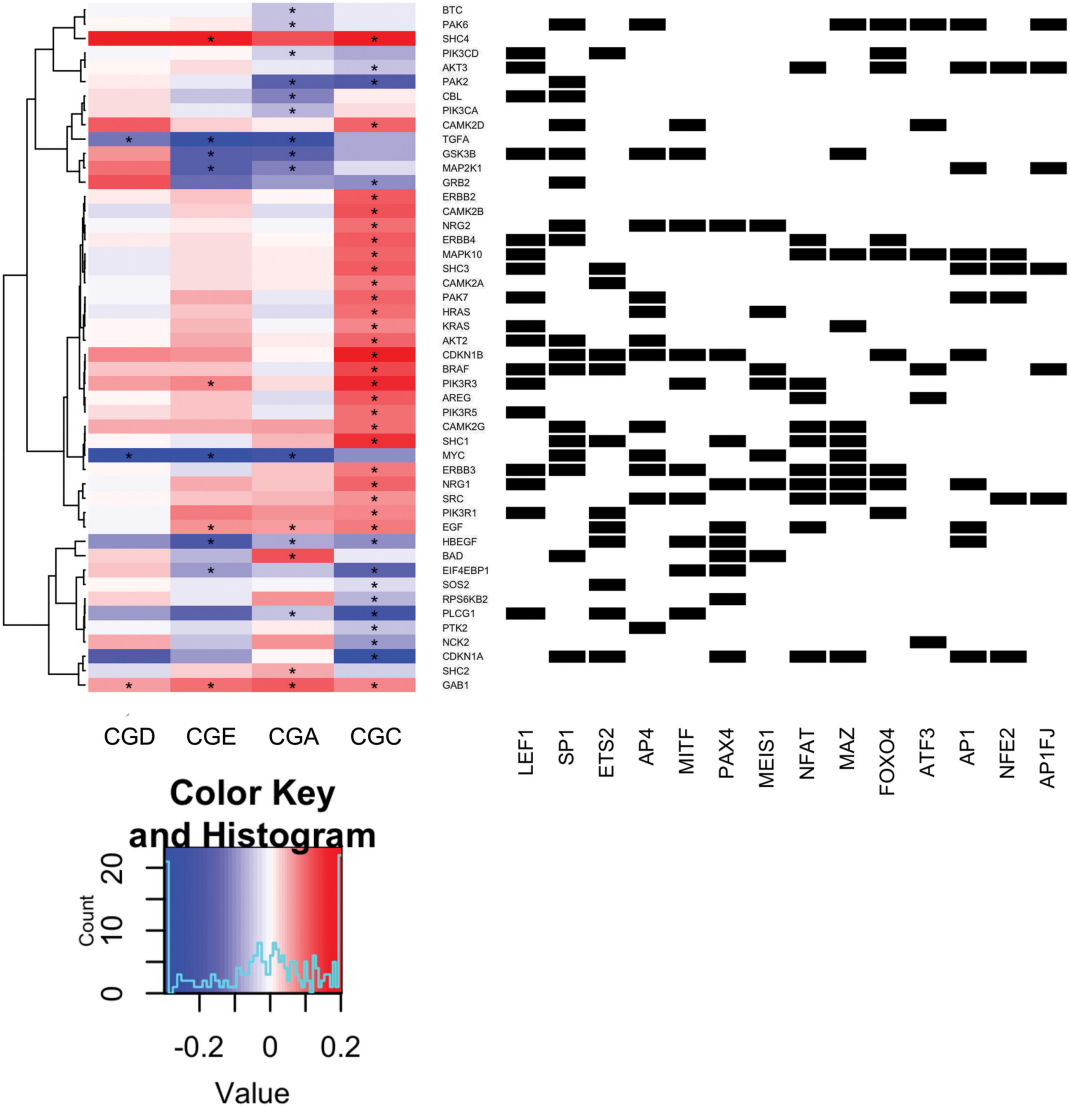
Transcription factors associated with ErbB pathway genes upregulated in combination sensitive cell lines treated with PLX4720. Unsupervised clustering of gene expression response (log_2_ fold change) to PLX4720 treatment (y-axis) of the ErbB signaling pathway genes of each cytotoxicity group (left heat map). Transcription factors (x-axis) that have binding motifs in the promoters of ERBB signaling pathway genes (black squares in binary matrix on right). Genes were selected for plotting based on passing the FDR corrected P Value threshold of 1% or better in at least one cytotoxicity group. Genes that demonstrated a FDR corrected P Value of 1% or better are denoted with an “*”.

We further analyzed transcription factor motifs that appear across multiple cytotoxicity groups in order to identify those that were more strongly enriched in CGC. For motifs that appeared in more than one group, we added the motif’s rank in CGC to the motif’s rank in the other group subtracted from 100. For motifs that appeared in more than the CGC and one other group, we calculated the arithmetic mean of the 100 minus the other groups’ ranks, and added that to the CGC rank. Using these averaged ranks summed with the rank for CGC, we found that CGC is more enriched than any other group for an additional 28 transcription factor binding motifs. We then found which of these transcription factors were associated with the promoters of ErbB signaling pathway genes. To visualize the ErbB pathway gene responses to PLX4720 treatment together with the transcription factors that putatively regulate them in one view, we plotted the log2 fold change of PLX4720 treated versus control of genes in the ErbB signaling pathway for each cytotoxicity group (Fig 5 and S9 Fig, Left) and identified transcription factors that have predicted binding motifs for those genes (Fig 5 and S9 Fig, Right). There is a subset of these factors closest to the heat map including *LEF1*, *SP1*, *AP4*, *ETS2*, and *PAX4* that putatively target multiple upregulated genes in the largest central cluster of the heat map. Additionally, ErbB family members contain binding motifs for *LEF1*, *SP1*, *AP4*, *NFAT*, *MAZ*, and *FOXO4* at their promoters. Notably, many of these factors have been associated with melanoma. *LEF1* has been shown to be highly expressed in proliferative cells in melanoma [40,41]. In a *HMGB1* knockdown in melanoma, a marked decrease in cell proliferation was observed mediated by *p21* in a *SP1* dependent manner [42]. *NFAT* has been identified as a potential drug target in melanoma using aminobisphosphonate zoledronic acid [43]. Lastly, *FOXO4* activation can induce senescence in *BRAF* ^*V600E*^ mutants [44]. Putting these results together, we find that transcription factors associated with MAPK signaling including ETS family members and co-factors along with factors that contain MAPK consensus sites [45] are apparently activated in response to PLX4720 treatment in combination sensitive cell lines to upregulate ErbB pathway genes, priming the cells for sensitivity to lapatinib.

### Reverse phase protein array analysis reveals down-regulation of oncogenic proteins across cytotoxicity groups eight hours after PLX4720 treatment

**Fig 6:**
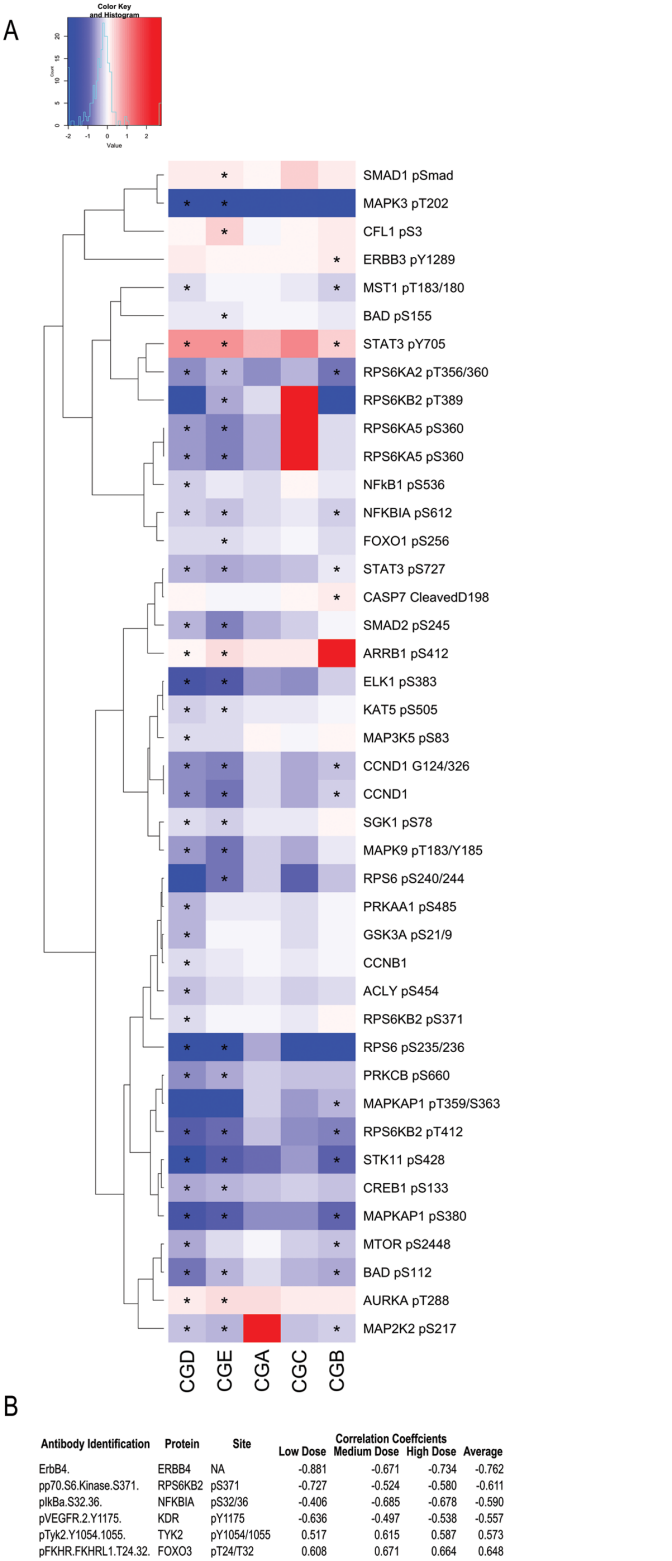
Reverse phase protein array reveals downregulation of RTK signaling pathways and a compensatory response of ErbB4 to PLX4720 treatment. Heat map of protein phosphorylation changes at 5% FDR across the four cytotoxicity groups. (A). Epitopes were selected for plotting based on passing the FDR corrected P Value threshold of 1% or better in at least one cytotoxicity group. Epitopes that demonstrated a FDR corrected P Value of 1% or better are denoted with an “*”. Correlating the response to treatment from the protein array with the cytotoxicity identified ErbB4 total protein fold changes as being the most anti correlated (B).

To test our hypothesis that resistant lines upregulate ErbB signaling activity, we performed reverse phase protein array analysis. We assayed approximately 132 protein epitopes across 105 different proteins sampling various oncogenic pathways including MAPK, PI3K, ErbB, etc. eight hours after initial treatment with PLX4720. We log2 transformed the normalized values, performed cytotoxic group-wise paired t-tests as described above and applied a 5% FDR cutoff. We identified 36 differentially expressed phosphoproteins, one cleavage site, and two proteins, whose total amount changed in response to PLX4720 treatment. Using these epitopes, we plotted their fold changes for each group due to treatment with PLX4720 in a heat map (Fig 6A). Surprisingly, as shown in Fig 6A, the lapatinib and combination resistant (CGD) and lapatinib resistant (CGE) groups display the strongest response to PLX4720 treatment with the majority of phosphoprotein levels going down. Notably, while not statistically significant by our stringent cutoff, the phosphoproteins in the other cytotoxicity groups displayed similar but weaker trends as those in the two resistant groups. This result together with the fact that we are assaying phosphoprotein levels eight hours after initial drug treatment may indicate that we are observing the direct downstream response of BRaf inhibition with minimal compensatory signaling being manifested at the level of protein phosphorylation. Interestingly, the weaker reduction observed at the phosphoprotein level in combination sensitive (CGC) and PLX4720 sensitive (CGA) cells may be due to the fact that the full compensatory signaling response requires transcription and translation [3]. This combination of a direct response of PLX4720 (i.e., downregulation) and delayed compensatory signaling may explain why we do not observe upregulation of proteins in the ErbB pathway 8 hours after PLX4720 treatment.

To see if there is a signature at the protein level indicating that RTK-dependent signaling was being weakly but consistently downregulated in PLX4720 sensitive cell lines and upregulated in PLX4720 resistant cell lines in response to PLX4720 treatment, we calculated the Pearson correlation coefficient between the normalized log2 fold change (PLX4720 treated over untreated cells) and cytotoxicity in response to PLX4720 treatment across cell lines (including DM331) for each of the three doses separately. We found the change in total *ERBB4* protein levels in response to PLX4720 treatment is the most anticorrelated epitope with PLX4720 cytotoxicity (Fig 6B; S9 Table). Other highly anticorrelated proteins were p70S6 kinase (p70S6K), the alpha inhibitor of *NFkB*, and *VEGFR*. The significantly differentially phosphorylated proteins in response to PLX4720 treatment and this correlation analysis suggest that PLX4720 inhibition may be broadly activating multiple RTKs beyond ErbB family members in CGC but reducing their activity in the other groups.

## Discussion

Inhibition of BRaf signaling as a therapy for the 50% of cutaneous melanomas that are mutant *BRAF* ^*V600E*^ is confounded by a wide variety of mechanisms of resistance, both intrinsic and acquired. Thus, there is considerable interest in understanding the general principles of resistance to BRaf inhibition so that druggable targets could be identified that would guide the development of effective drug combinations. In a high throughput screen, we found that the combination of the mutant BRaf inhibitor PLX4720 with the ErbB family inhibitor lapatinib caused synergistic cytotoxicity in the six lines of our 12 cell-line panel that showed the greatest intrinsic resistance to BRaf inhibition. By performing hierarchical clustering of the cytotoxicity dose-response data, we identified five different cytotoxicity groupings, reflecting extraordinary heterogeneity in biological responses to drug treatment in this panel. Novel results from our systems-level molecular profiling analysis include linking lineage (reflected by *MITF* expression), *BRAF* zygosity, cell-line specific mutations in proto-oncogenes and tumor suppressors and widespread transcriptional upregulation of ErbB family members and other receptor tyrosine kinases in the response and resistance of melanoma cells to PLX4720. Examination of the transcriptional responses to PLX4720 treatment revealed the involvement of transcription factors with the potential to be responsive to the MAPK pathway, providing a possible convergent mechanism underlying the diversity of phenotypic responses.

### Analysis of the basal state: cytotoxicity, transcriptome and genome

Principal Component Analysis of the basal (untreated with drugs) transcriptomes of the five cytotoxicity groups revealed a correlation between drug synergy and the second Principal Component. The strongest transcriptional correlation and anticorrelation to synergy was among genes encoding cell surface receptors including GPCRs and neuroactive ligand signaling GPCRs. Many GPCRs are capable of activating the MAPK pathway, either directly or by transactivating RTKs utilizing neurotropin [46–48], thus identifying a potential mechanism for bypassing BRaf inhibition. Nevertheless, the broad and diverse genes and pathways did not yield clear, concrete mechanistic insights into drug response. The first Principal Component of the basal transcriptomic analysis correlated with *MITF* expression, which has been implicated by several reports in drug resistance and oncogenesis in melanoma [9,19,49]. For example, Konieczkowski et al. recently identified high *MITF* levels and *MITF* transcriptional target activity as associated with increased sensitivity to PLX4720 treatment [12]. However, in our panel we obtained a very modest association between *MITF* and IC50 for PLX4720, an association that was driven almost entirely by our single most resistant cell line; when this line (DM331) was dropped from the analysis, no association with *MITF* appeared. However the study by Konieczkowski et al. [12] utilized 29 cell lines, many of which displayed lower *MITF* expression levels than occurred in our panel; *MITF* gene expression levels would be classified as *MITF* high for all our cell lines according to the criteria established by Muller et al. [13]. Thus, the Konieczkowski study, by dint of range of *MITF* expression and number of cell lines, was better powered to detect a modest association.

The groupings of our cell lines according to cytotoxic response to drug treatment and basal expression profile can be rationalized in part by *BRAF* ^*V600E*^ zygosity status. The high proportion of homozygous *BRAF* mutants in our study (4 out of 12) helps to highlight this tendency, and was strengthened by comparison with data in the Cell Line Encyclopedia. As shown in S1 Table and S4 Fig, cell lines (excluding SKMEL28 and SKMEL24) on the left of the PCA plot contain heterozygous *BRAF* ^*V600E*^ mutations. Regarding cytotoxic response to vemurafenib treatment, the cell lines tend to be more resistant on the left. Additionally, the cell lines on the top of the PCA plot respond synergistically to the combination of vemurafenib and lapatinib while those on the bottom do not. It has been shown that vemurafenib binding to *BRAF* ^*V600E*^ paradoxically activates downstream MAPK signaling via dimerization with non-mutant RAF family members and allosteric activation of the non-mutant partner [50]. In a homozygous *BRAF* ^*V600E*^ cell line, opportunities for this paradoxical activation are limited to the heterodimers with *RAF1*, which could partially explain increased dependency on the output of the mutationally activated BRaf.

Our results support the findings of Konieczkowski et al. [12] that overexpression of *BRAF* ^*V600E*^ suppresses *MITF* gene expression: in our cell lines, *MITF* levels tend to be lower in homozygous versus heterozygous lines. We hypothesize that *BRAF* ^*V600E*^ levels are inhibiting *MITF* levels, which in that sense may be acting more as a passenger. Nevertheless, *MITF* is a potent regulator of melanoma lineage and to a large extent determines the basal transcriptional profile, hence its association with the first Principal Component of our basal transcriptional analysis.

Layered over and interacting with *BRAF* ^*V600E*^ zygosity and *MITF* levels in affecting sensitivity to PLX4720, are mutations in additional oncogenes and tumor suppressor genes. Mutations in cell surface proteins and RTKs potentially explain **(1)** cytotoxicity group-*BRAF* zygosity exceptions including SKMEL28 (homozygous *BRAF* yet combination sensitive) and SKMEL24 (heterozygous *BRAF* yet PLX4720 sensitive), **(2)** lapatinib resistant cell lines, which are heterozygous *BRAF* and **(3)** DM331, which is an outlier from a cytotoxic and transcriptional profile grouping perspective. **(1)** In SKMEL28, the most striking variant was the *EGFR* exception mutation, which has previously been identified as a gain of function mutation [51,52] and could explain this line’s resistance to PLX4720 treatment despite being homozygous for *BRAF* ^*V600E*^. In SKMEL24 we found a number of potentially damaging mutations in cell surface proteins including *TNFRSF14*, *ERBB3* and *EGFR*, which could limit SKMEL24’s ability to activate alternative growth pathways in the presence of BRaf inhibition, thereby, potentially explaining its sensitivity to PLX4720 treatment despite its heterozygous *BRAF* status. The *ERBB3* mutation is particularly interesting given that ErbB family members are lapatinib targets; however, this mutation was not found to be damaging by polyphen2 analysis and would require more study to determine if it alters *ERBB3* function. **(2)** Both lapatinib resistant cell lines contained a damaging mutation in *ALK*, which is a receptor tyrosine kinase that has been previously associated with multiple cancer types [8,20,29] and is an activating mutation in many types of lung cancer [8]. We hypothesize that the *ALK* mutation could be stimulating growth, thereby explaining why the cells in the secondary resistant group are resistant to lapatinib. **(3)** DM331 is an exception in that it is homozygous for *BRAF* ^*V600E*^ yet it is by far the most resistant cell line to PLX4720 treatment. Additionally, its expression profile at the basal state most closely associates it with A375, one of the most sensitive lines. However, A375 resistant lines have been generated multiple times, indicating that its sensitivity is not immutable [10,18]. We find *NRG1*, an ErbB family ligand is highly upregulated in DM331 only, and not in A375. On the other hand, addition of *NRG1* to A375 causes resistance to PLX4720 [18]. Combined with the robust expression of *EGFR* in DM331 this may explain the profound resistance of DM331 to BRaf inhibition.

### Adaptive responses to BRaf inhibition

We observed broad upregulation of ErbB pathway gene expression 8 hours after PLX4720 treatment, only in the group of cell lines that were sensitive to the combination of PLX4720 and lapatinib. This result is consistent with that of other groups that have found *ERBB3* [18,21,53] and other RTKs [12,13] upregulated in BRAF^V600E^ melanoma in response to PLX4720 treatment.

Surprisingly, we observed a relatively strong downregulation of oncogenic phosphoprotein levels after 8 hours of PLX4720 treatment in CGD and CGE cell lines with a similar but much weaker downregulation of the same proteins in CGA and CGC. We hypothesize that the downregulation observed at the protein level is the direct result of PLX4720 treatment followed by delayed, compensatory signaling in the combination and PLX4720 sensitive cell lines. As partial confirmation of this, we found that *ERBB4* total protein and *VEGFR* phosphorylation levels were anticorrelated with PLX4720 cytotoxic response across cell lines. This indicates that treatment with PLX4720 induces receptor tyrosine kinase expression, most likely as a compensatory survival mechanism in lines resistant to treatment as demonstrated in other studies [18,53]. This also potentially explains the synergy between PLX4720 and lapatinib that we observe in a subset of cell lines. Not only is ErbB family inhibition effective via lapatinib, but inhibition of other receptor tyrosine kinases, via masitinib was also an effective inhibitor based on our drug screen (Roller et al. In preparation). It is possible that a “threshold” of RTK activity needs to be reached in order to generate resistance, perhaps by activating *NRAS* signaling and MAPK pathway reactivation.

Lastly, it appears that PLX4720 treatment potentially activates ETS family transcription factors and co-factors to upregulate ErbB pathway genes as well as those of other receptor tyrosine kinase signaling pathways. Abel et al. observed [18] *ERBB3* activation due to vemurafenib treatment; however they saw activation via *FOXD3*. Chandarlapaty et al. [54] identified *ERBB3* activation in response to *AKT* inhibition via phosphorylated *FOXO3* in cancer cell lines from multiple tumor types, which were resistant to *AKT* inhibition. However, the correlation between changes in phosphorylated *FOXO3* induced by PLX4720 and PLX4720 cytotoxicity in our melanoma lines indicate that *FOXO3* phosphorylation is increased in the most sensitive lines suggesting alternative regulatory programs in the *BRAF* ^*V600E*^ melanoma cell lines that we studied. If *FOXO3* phosphorylation activates *ERBB3* as a compensatory mechanism to BRaf inhibition, we would expect its log2 fold change to be anticorrelated with cytotoxic response to PLX4720, similar to the response of *ERBB4*. We do see *FOXO4* among the transcription factors whose motifs are enriched at the promoters of upregulated ERBB pathway genes. Importantly, while previous studies have observed *ERBB3* upregulation in response to various inhibitors [18,24,54], we find broad upregulation of ErbB family members in response to PLX4720 treatment.

It has been noted that multiple receptor tyrosine kinases confer resistance to many forms of treatment [55]. We find that inhibition of BRaf in melanoma cell lines leads to compensatory signaling via ErbB family members, which are RTKs. Consequently, we and others [21] find the drug combination vemurafenib and lapatinib to be highly synergistic in a subset of *BRAF* ^*V600E*^ melanoma cell lines. Additionally, we have observed that inhibition of other RTKs, also elicits a synergistic response when combined with BRaf inhibition. Taken together, our results suggest RTK mediated signaling is an adaptive response to mutant BRaf inhibition in melanomas and suggests combination therapy with specific RTK inhibitors to have potential clinical benefit.

## Methods

### Cell culture

Melanoma cell lines used in this study were derived from tumors taken from tumor-involved lymph nodes from patients at the University of Virginia (VMM18, VMM5A, VMM15, VMM17, and VMM12) or from patients at Duke University (DM6, DM13, and DM331) as described previously [56–60]. A375, SKMEL28, SKMEL24 and HT144 were obtained from ATCC. All lines were propagated in RPMI-1640 (Invitrogen) supplemented with 5% or 10% FBS (Gemini Bio-Products).

### Cytotoxicity assays

Four hours after being plated in 96-well plates, cells were treated with inhibitors or vehicle control in phenol red–free RPMI-1640 (Invitrogen) without FBS and incubated for 3 days at 37°C. alamarBlue (Invitrogen) was added to wells and incubated for 4 hours at 37°C. Fluorescence was measured at 560 nm excitation/590 nm emission on a Synergy 2 plate reader (BioTek Instruments). Mean results and SE were calculated for triplicate samples.

Cell Culture and Generation of Transcriptome, Proteome and Exome Data.

### Gene array

Cells were plated and incubated overnight before being treated, in duplicate, with inhibitors or vehicle control in phenol red–free RPMI-1640 without FBS for 8 hours at 37°C. Cells were placed on ice and rinsed with cold 1× PBS. Cells were collected and RNA was isolated using the Qiashredder (Qiagen) and RNeasy Mini Kit (Qiagen). RNA was quantified on the NanoDrop 2000 spectrophotometer (Thermo Scientific) and RNA quality was inspected on a 1% agarose gel. Biotin labeled RNA was hybridized to Illumina 3′IVT human HT-12 BeadChip arrays.

### Reverse phase protein array

Cells were plated in p60 dishes and incubated overnight to allow the cells to adhere before being treated with inhibitors or vehicle control in phenol red-free RPMI Medium 1640 without fetal bovine serum for 8 hours. Cells were washed and lysed in 1:1 2x Sample Buffer:Tissue Extraction Reagent (T-PER) (Life Technologies). Following lysis the samples were sonicated and centrifuged to clear. Samples were diluted to requested concentration and sent to the lab of Emanuel (Chip) Petricoin at George Mason University for Reverse Phase Array Analysis. Raw sequencing reads were generated using a combination of Nimblegen v2 and Agilent v1 capture probes.

### Exome sequencing

Cells were grown to 80% confluent, trypsinized, and collected in normal growth media. Cells were centrifuged to pellet, wash with PBS, and centrifuged a second time. DNA was extracted using the DNeasy Blood and Tissue Kit (Qiagen) and quantified on the Nanodrop 2000 spectrophotometer (Thermo Scientific). Exome sequencing was performed by Hudson Alpha Institute for Biotechnology (Huntsville, Alabama).

### Statistical analysis of transcriptome and proteome data

Illumina microarray data was variance stabilized transformed [61,62] using the *lumi* Bioconductor package in R [63]. Genes that displayed significant variation in basal gene expression across all twelve cell lines were identified using *limma* to perform one way ANOVA and derive Benjamini-Hochberg False Discovery Rate (FDR) adjusted p-values [64,65] and applying a 0.1% FDR threshold. Significantly varying genes and cell lines were clustered using the R package *pvclust* [66] with the Pearson correlation distance measure and average linkage (Fig 2A). We assessed the significance of the clusters by performing 1000 iterations of the clustering introducing random variations and assessing how much randomness was required to loose a specific branch. Cell lines were also clustered according to cytotoxic response to 125 nM, 625 nM and 1250 nM of PLX4720 and 1000 nM, 2000 nM and 4000 nM of lapatinib and a three-by-three dose matrix of the two drugs. We used *pvclust* with a correlation distance measure and average linkage (Fig 3A). We identified differentially expressed genes (upregulated or downregulated) within each cytotoxicity group of cell lines in response to PLX4720 treatment using *limma* to perform paired t-tests whereby untreated and PLX4720 treated samples were paired across all replicates and cell lines within a group and applying a 1% FDR cutoff. For genes with multiple probes, we selected the one with the lowest FDR. Normalized log2 reverse phase protein array (RPPA) data was generated using methods described in [62]. We performed a paired t-test analysis described above (for gene expression data) to identify differential (untreated versus PLX4720 treated within cytotoxicity group) protein levels (upregulated or downregulated) by applying a 5% FDR cutoff. Log2 fold change of proteins in PLX4720 treated versus untreated cells were then correlated to cytotoxic response of cell lines to each dose of PLX4720 using the Spearman correlation coefficient. We summarized the three correlation coefficients across doses derived for each protein by calculating the average value.

### Statistical analysis of exome data

Exome probes were genotyped using the Genome Analysis Toolkit and the HG19 build as a reference genome. Single nucleotide variants were annotated by *SeattleSeq*. Genes with novel variants were identified by removing variants previously identified in dbSNP, or 1000Genomes. Novel variants were further filtered by selecting only nonsense and missense mutations as identified by *SeattleSeq*. The list of genes containing novel nonsynonymous variants were selected by retaining only those that were identified by [20] as being significant drivers across multiple cancers. The list of novel, nonsynonymous variants located in these genes were input to *PolyPhen2* to determine the potential functional severity of these nonsynonymous variants. Genes were identified that uniquely contained variants in each cell line, and within each cytotoxicity group.

### Pathway enrichment analysis

Gene sets found to be significant underwent pathway enrichment analysis via Pathway Express and Gene Set Enrichment Analysis (GSEA) using MSigDB datasets. Significant gene sets and pathways were identified as having a FDR enrichment value of 0.05 for both tools. To ascertain significance of transcription factor binding motifs, we identified motifs unique to the CGC. For motifs not unique to the CGC, we summed the rank of CGC with 100 minus the rank of the other group. When multiple groups contained the motifs, we averaged the ranks, then summed the rank of CGC with 100 minus the averaged rank.

## Supporting Information

S1 Fig Synergy scores for melanoma cell lines. Synergy score (left y-axis) across all 12 cell lines (bottom x—axis) for escalating doses of lapatinib (top x-axis) and PLX4720 (right y-axis). Synergy Score was calculated using the Bliss model of independence. Synergy Score = C_Combination_ − (1 – (1 – C_Secondary Drug_)*(1 – C_Primary Drug_)) where C_Combination_ is the observed cytotoxicity of the combination treatment, C_Secondary Drug_ is the observed cytotoxicity of the secondary drug, and C_Primary Drug_ is the observed cytotoxicity of the primary drug.
(EPS)

S2 Fig *MITF* ChIP-Seq targets overlapping Cluster IV genes. Number of overlapping genes (purple) between *MITF* ChIP-Seq binding sites (blue) and Cluster IV gene list (pink).
(EPS)

S3 Fig Melanoma cell lines segregate according to their cytotoxicity groups and *MITF* expression levels in principal component analysis plot. Basal PCA plot with cytotoxicity groups encircled, and homozygous *BRAF* mutants denoted with a “*” (A). Relative expression in Log2 of *AXL* (Red) and *MITF* (Blue) (B).
(EPS)

S4 Fig Cytotoxic responses of melanoma cell lines to single and combination treatments. Box plots of cytotoxic response to single drug treatment (top 2 rows) and escalating PLX4720 doses of the combination drug treatment (bottom 3 rows). Doses along the bottom refer to either the single drug treatment (top 2 rows), or the secondary drug treatment dose for the combination drug treatment (bottom 3 rows). Red dots denote negative cytotoxic responses, and blue dots denote positive cytotoxic responses.
(EPS)

S5 Fig Transcriptional response of genes in the ErbB signaling pathway to PLX4720 treatment for each cytotoxicity group. The responses for each group are CGD (top left), CGE (top right), CGA (bottom left), and CGC (bottom right). Nodes in red denote an upregulation, nodes in blue denote a downregulation. Refer to S6 Table for fold change values used to generate pathway diagram.
(EPS)

S6 Fig Transcriptional response of genes in the pathways in cancer pathway response to PLX4720 treatment for each cytotoxicity group. The responses for each group are CGD (top left), CGE (top right), CGA (bottom left), and CGC (bottom right). Nodes in red denote an upregulation, nodes in blue denote a downregulation. Refer to S6 Table for fold change values used to generate pathway diagram.
(EPS)

S7 Fig Transcriptional response of genes in the Wnt signaling pathway response to PLX4720 treatment for each cytotoxicity group. The responses for each group are CGD (top left), CGE (top right), CGA (bottom left), and CGC (bottom right). Nodes in red denote an upregulation, nodes in blue denote a downregulation. Refer to S6 Table for fold change values used to generate pathway diagram.
(EPS)

S8 Fig Transcriptional response of genes in the MAPK signaling pathway response to PLX4720 treatment for each cytotoxicity group. The responses for each group are CGD (top left), CGE (top right), CGA (bottom left), and CGC (bottom right). Nodes in red denote an upregulation, nodes in blue denote a downregulation. Refer to S6 Table for fold change values used to generate pathway diagram.
(EPS)

S9 Fig Transcription factor motifs associated with ErbB pathway gene expression response across melanoma cell lines treated with PLX4720. Unsupervised clustering of gene expression response to PLX4720 treatment (y-axis) of the ErbB signaling pathway of each cell line (Left). Genes were selected for plotting based on passing the FDR corrected P Value threshold of 1% or better in at least one cytotoxicity group. Genes that demonstrated a FDR corrected P Value of 1% or better for the t-test for an individual cell line are denoted with an “*”. Transcription factors were identified that have binding motifs in the promoters of these genes (Right).
(EPS)

S1 Table Transcription factor enrichment results of one way ANOVA clusters III and IV. Clusters III and IV were identified from the one way ANOVA analysis (Fig 2A). Each cluster was submitted to MSigDB using the transcription factor gene set to identify transcription factors that may be controlling the expression of genes present in clusters III and IV.
(XLSX)

S2 Table First principal component correlation probe list. List of probes from gene expression microarray that demonstrated a correlation coefficient greater than 0.8 or less than -0.8 with the loadings of the first principal component.
(XLSX)

S3 Table Second principal component correlation probe list. List of probes from gene expression microarray that demonstrated a correlation coefficient greater than 0.8 or less than -0.8 with the loadings of the second principal component.
(XLSX)

S4 Table Principal component correlation MSigDB enrichment results. Probes that demonstrated significant correlation (S2 and S3 Tables) underwent a hypergeometric enrichment analysis via MSigDB using the canonical pathways gene set.
(XLSX)

S5 Table Polyphen2 results of enriched novel nonsynonymous variants. Variants that were present in individual cell lines of interest or present in all cell lines of a cytotoxicity group were submitted to Polyphen2 to ascertain potential functional significance.
(XLSX)

S6 Table Paired T test differential expression analysis results for all genes. Log2 Fold changes, p values, and Bonferroni corrected p values (FDR) for differential expression analysis of each cytotoxicity group.
(XLSX)

S7 Table Pathway Express results for each cytotoxicity group. Differentially expressed genes for each cytotoxicity group that passed a FDR threshold of 1% or better were submitted to Pathway Express to identify pathways significantly affected by treatment.
(XLSX)

S8 Table MSigDB results of transcription factor enrichments and pathway enrichments for each cytotoxicity group. Differentially expressed genes for each cytotoxicity group that passed a FDR threshold of 1% or better were submitted to MSigDB to identify pathways and transcription factors significantly affected by treatment.
(XLSX)

S9 Table Reverse phase protein microarray PLX4720 response cytotoxicity correlation. Table of correlations between log2 fold changes of cell lines’ protein responses to treatment of PLX4720 with cell lines’ cytotoxic responses to PLX4720.
(XLSX)
